# m6A modification in allergic airway diseases: a narrative review from a cellular perspective

**DOI:** 10.3389/fimmu.2026.1943431

**Published:** 2026-10-21

**Authors:** Leilei Zheng, Siyu Duan, Ziyi Jia, Zhihan Xu, Yisha Wu, Shikai Han, Jinmei Xue

**Affiliations:** 1 Department of Otolaryngology, Head and Neck Surgery, the Second Hospital, Shanxi Medical University, Taiyuan, Shanxi, China; 2 Shanxi Key Laboratory of Rapid Diagnosis and Precision Treatment of Airway Allergic Diseases, Taiyuan, Shanxi, China; 3 Shanxi Medical University, Taiyuan, Shanxi, China

**Keywords:** allergic airway diseases, allergic asthma, allergic rhinitis, epigenetics, epithelial-immune crosstalk, m6A modification

## Abstract

N6-methyladenosine (m6A) represents the most prevalent modification on eukaryotic RNA and plays a critical role in nearly all stages of RNA expression regulation. Extensive research demonstrates that the dysregulation of m6A modification plays a significant role in the disruption of epithelial barriers, the induction of oxidative stress, the activation of inflammatory cells, and the remodeling of airways, all of which are characteristic pathological features of allergic airway diseases (AADs). Given the distinct roles of various cell types in the progression and resolution of AADs, understanding how m6A functions across cellular contexts is essential for a comprehensive understanding of the disease’s pathological mechanisms. This review begins by outlining the fundamental biological characteristics of m6A modification. Subsequently, we explore how dynamic m6A modifications affect AADs progression from the perspectives of airway epithelial cells, innate immune cells, and adaptive immune cells, emphasizing the pivotal regulatory role of m6A in epithelial-immune crosstalk. We then summarize how cellular context shapes the functional heterogeneity of m6A modification and discuss the potential underlying mechanisms. Finally, we summarize the latest progress in the use of m6A-related enzymes as tools for disease prediction, therapeutic intervention, and evaluation of allergen immunotherapy efficacy, and discuss the opportunities and challenges associated with the clinical translation of m6A-targeted therapeutic strategies.

## 1 Introduction

Allergic airway diseases (AADs) include allergic rhinitis (AR) and allergic asthma (AA). It is estimated that nearly 500 million and 300 million people worldwide suffer from AR and AA, respectively, and the prevalence of these conditions continues to rise (1, 2). The pathogenesis of AADs is highly complex, involving a multitude of cell types and immunoregulatory processes (3, 4). Alterations in epithelial cells are pivotal in initiating the inflammatory cascade (5). Allergen exposure induces airway epithelial cells to secrete various inflammatory mediators, known as epithelial alarmins, which recruit immune cells and compromise the epithelial barrier’s integrity, thereby perpetuating type 2 inflammation and airway damage (6–8). Subsequently, innate immune cells (9–11), such as dendritic cells (DCs) and Group 2 innate lymphoid cells (ILC2s), facilitate adaptive T-cell (12, 13) and B-cell (14) immunity either directly or indirectly. Therefore, the distinct and coordinated contributions of multiple cell types are critical to the pathophysiology of AADs. Currently, a variety of anti-airway allergy drugs are extensively utilized in clinical practice. However, patient responses to these treatments exhibit considerable variability (2, 15). Such heterogeneity may reflect differences in cellular states, inflammatory pathways, and tissue microenvironments across individuals and disease contexts. A better understanding of the molecular mechanisms operating within distinct cellular contexts and their interactions is therefore essential for elucidating AADs pathogenesis and developing more effective therapeutic strategies.

Epigenetic mechanisms play a pivotal role in governing the differential trajectories and functional evolution of various cell types involved in allergic airway inflammation by influencing the splicing, translation, localization, and transport of modified RNAs (16–18). Among these mechanisms, N6-methyladenosine (m6A) methylation emerges as a critical epigenetic modification in the pathogenesis of AADs. For instance, Han et al. showed that METTL3 deficiency in macrophages enhances YTHDF3-dependent degradation of PTX3 mRNA, thereby promoting the activation of M2 macrophages and triggering airway allergic symptoms (19). In epithelial cells, the m6A demethylase Fat mass and obesity-associated protein (FTO) mitigates allergic inflammation by modulating the nuclear factor kappa B (NF-κB) and epithelial-mesenchymal transition (EMT) signaling pathways (20). METTL3 facilitates the degradation of SOX5, thereby inhibiting T helper 2 (Th2) cell differentiation through an epithelial-immune crosstalk mechanism (21). Consequently, prior to the development of m6A-targeted therapies, it is imperative to concentrate on the regulatory mechanisms of m6A modulators at the single-cell level and within epithelial-immune interactions in AADs.

This review first provides an overview of m6A RNA methylation. We then focus on pathophysiological mechanisms of m6A regulation across various cell types in AADs, aiming to deepen our understanding of AADs’ complex pathogenesis from a cellular perspective. In addition, we summarize the current evidence for context-dependent m6A regulation in AADs. Finally, we discuss the clinical significance and limitations of m6A-based biomarkers and therapeutic strategies. Future studies integrating multiple technologies, such as single-cell epitranscriptomics and targeted delivery systems, will be essential for developing m6A-targeted interventions for AADs.

## 2 Scope and literature search

We searched for English-language literature published between 2021 and June 1, 2026. Additionally, we consulted published reviews, meta-analyses, and other sources to supplement the search. **Table 1** presents the number of included studies involving human subjects, animal models, and cell lines.

**Table 1:** The role of m6A modification in airway epithelial cells.

Study	Disease	Cell type	m6A regulator	Target gene/pathway	Biological function	Key evidence
(39)	AR	Bronchial epithelial cells	RBM15B, METTL3/METTL14	CHOP	Inhibiting AR-induced endoplasmic reticulum stress and ameliorating nasal epithelial barrier damage	OVA-induced AR mouse model, OVA-induced HNEpC cells
(40)	AR	Bronchial epithelial cells	METTL3, IGF2BP1	circMIRLET7BHG, ADAM10	The METTL3/circMIRLET7BHG/ADAM10 axis disrupts the nasal mucosal epithelial barrier.	Human nasal mucosa tissue, OVA-induced AR mouse model, OVA-induced HNEpCs
(41)	AR	Bronchial epithelial cells	METTL3, YTHDC1, IGF2BP2	circCDKAL1	METTL3-mediated m6A modification of circCDKAL1 impairs nasal epithelial cell adhesion via the IGF2BP2/JARID2/HMGB1 axis.	Human nasal mucosa tissue, OVA-induced AR mouse model, OVA-induced HNEpCs
(42)	AR	Nasal epithelial cells	METTL3	PTBP1	The METTL3/PTBP1/TXNIP molecular axis causes nasal epithelial barrier dysfunction by regulating oxidative stress and ferroptosis	OVA-induced AR mouse model, IL-13-stimulated HNEpCs
(43)	AA	Bronchial epithelial cells	METTL3	GPX4	Overexpression of METTL3 enhances the viability and proliferative capacity of bronchial epithelial cells.	OVA-induced AA mouse model, IL-13-stimulated HNEpCs
(44)	AR	Nasal epithelial cells	WTAP	FBXW7	Inhibition of WTAP expression alleviates IL-13-induced oxidative stress injury and apoptosis in hNECs	IL-13-stimulated HNEpCs
(48)	AA	Bronchial epithelial cells	FTO	IKBKB	PM2.5 exposure upregulates FTO expression and exacerbates epithelial barrier damage	HDM-induced AA mouse model, PM2.5-treated Beas-2B cells
(49)	AA	airway epithelium	FTO	FOXJ1	FTO stabilizes FOXJ1 mRNA in an m6A-dependent manner, promoting airway ciliary motility.	OVA-induced AA mouse model, Primary human bronchial epithelial cell
(50)	AA	Bronchial epithelial cells	METTL3, YTHDF2	STC2	PM2.5 exposure significantly increased the level of m6A methylation in STC2 mRNA, leading to the activation of mitophagy and the exacerbation of asthma	Peripheral blood samples from asthma patients, HDM-induced AA mouse model, PM2.5-treated Beas-2B cells
(53)	AA	Bronchial epithelial cells	METTL3, YTHDF3	COX4I1	Under PM2.5 exposure, exosomal lncRNA PAET inhibits oxidative phosphorylation by regulating the METTL3-YTHDF3-COX4I1 axis, thereby causing DNA damage	Pediatric plasma, human bronchial epithelial cells
(55)	AA	Bronchial epithelial cells	FTO, IGF2BP1/2/3	ACKR3	PAHs enhance NF-κB signaling and autophagy via the FTO-ACKR3 axis	HDM/PAHs-induced AA mouse model, Beas-2B cells
(56)	AA	Alveolar epithelial cells and bronchial epithelial cells	METTL3, YTHDF1	CLOCK	YTHDF1-m6A-METTL3 promotes the expression of NLRP3 and IL-1 via the target gene CLOCK, thereby stimulating the airway inflammatory response	HDM-induced AA mouse model, Primary human alveolar epithelial cells and BEAS-2B cells treated with HDM or OVA
(59)	AA	Bronchial epithelial cells	METTL14	MEK/ERK signaling pathway	METTL14 overexpression reverses EMT, cell proliferation, and migration induced by ETS1 or RBPJ deficiency.	OVA-induced AA mouse model, Recombinant human TGF-β1-treated Beas-2B cells
(60)	AA	Bronchial epithelial cells	METTL3	miR-143-3p	METTL3 inhibits airway epithelial cell migration and EMT by upregulating miR-143-3p expression, thereby reducing airway remodeling	TGF-β1-treated human bronchial epithelial 16HBE cells
(20)	AA	Bronchial epithelial cells	FTO	Transcripts of extracellular matrix proteins and adhesion molecules	The demethylase activity of FTO is involved in the regulation of NF-κB and EMT-related pathways.	IL-4/IL-13-treated BEAS-2B cells, Mouse model of HDM-induced allergic airway inflammation

## 3 Overview of m6A RNA modification

m6A RNA methylation is recognized as the most prevalent and evolutionarily conserved post-transcriptional modification in mammalian mRNA and non-coding RNA (22–24). This modification is governed by three distinct protein families: m6A methyltransferases (writing enzymes), m6A demethylases (erasing enzymes), and m6A-binding proteins (reading enzymes), which collectively influence RNA splicing, nuclear export, stability, degradation, and translation (22, 25, 26).

The m6A modification process mainly relies on the methyltransferase complex (MTC), with the METTL3/METTL14 heterodimer acting as a central component (27). Several auxiliary subunits are used to locate and initiate methylation, including Wilms tumor 1-associated protein (WTAP), RNA-binding motif protein 15 (RBM15), Zinc finger-CCCH domain-containing protein 13 (ZC3H13), and VIRMA (28–30). Methyltransferases generally target the conserved RNA sequence RRACH, which is predominantly located in the 3’ untranslated region (UTR), extended exons, and regions proximal to the stop codon (31). m6A demethylases, comprising the enzymes FTO and ALKBH5, recognize and remove m6A modifications within RNA (32, 33). m6A-binding proteins facilitate the interaction of m6A-modified RNA with mRNA processing enzymes, encompassing the YTH-domain protein family, the IGF2BP family, and proteins of the heterogeneous ribonucleoprotein family (34–36).

## 4 Dysregulation of m6A in allergic airway diseases

### 4.1 Airway epithelial cells

Not only do airway epithelial cells form the body’s primary physical barrier against environmental stimuli, but they also play a vital role in immune signaling and tissue repair. In AADs, airway epithelial cells maintain tissue integrity through intercellular adhesion complexes between neighboring epithelial cells and regulate the immune microenvironment by secreting cytokines, chemokines, and epithelial-derived alarmins (37, 38). In light of this, it is imperative to elucidate how m6A modifications can reverse epithelial dysfunction by regulating a series of molecular mechanisms within airway epithelial cells (see **Figure 1** and **Table 1**).

**Figure 1:**
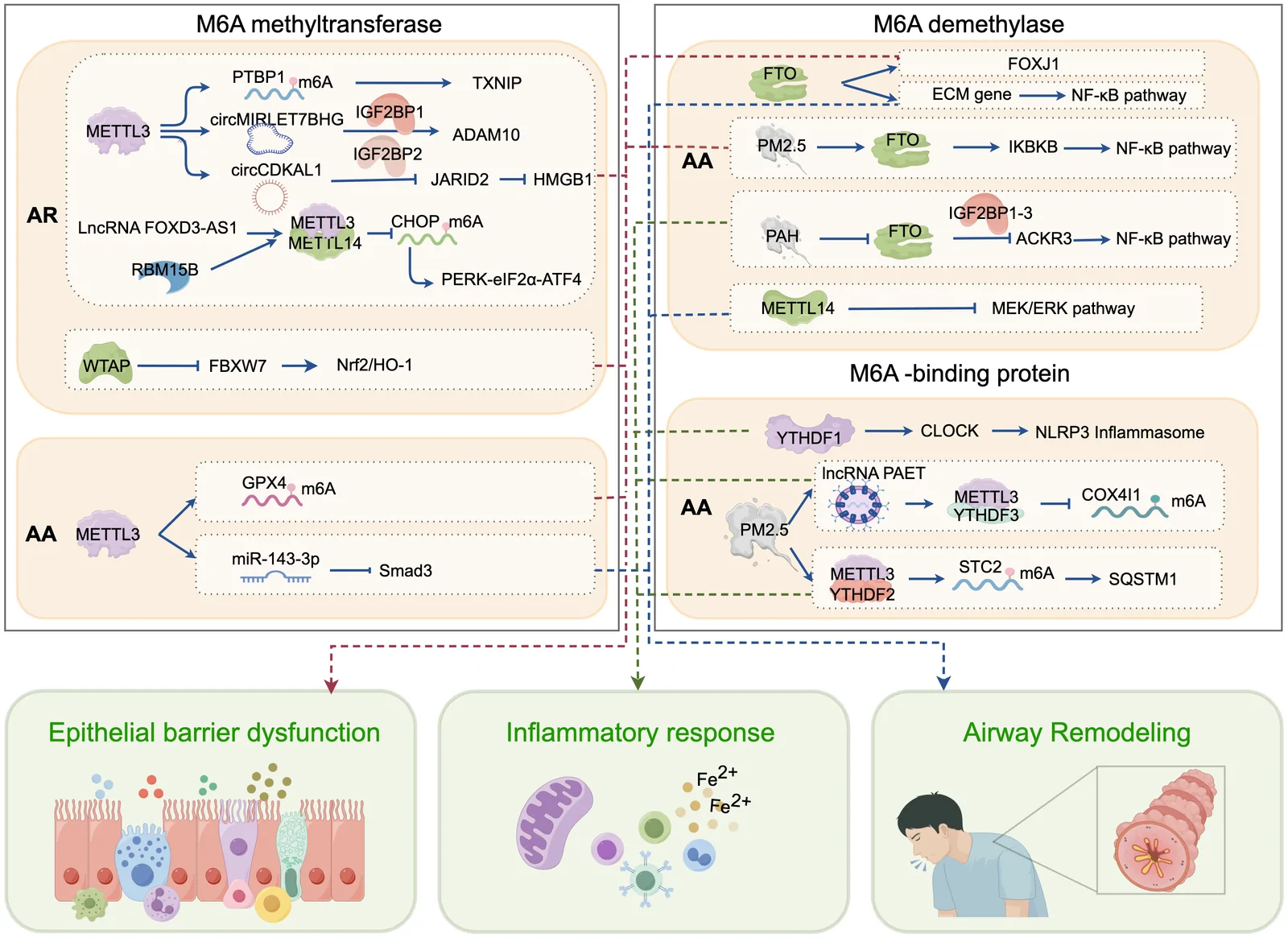
The role of m6A modification in airway epithelial cells. Abnormal m6A methylation contributes to epithelial barrier dysfunction, the inflammatory response, and airway remodeling through the regulation of various signaling pathways. Arrows indicate positive regulatory effects (sharp), inhibitory effects (blunt). m6A, N6-methyladenosine; AR, Allergic Rhinitis; AA, Allergic Asthma; ECM, extracellular matrix; PM2.5, Particulate Matter with aerodynamic diameter ≤ 2.5 μm; PAH, polycyclic aromatic hydrocarbons.

#### 4.1.1 Epithelial barrier integrity disruption

The airway epithelial barrier functions as the primary defense mechanism against inhaled allergens and environmental challenges, with its structural and functional integrity being essential for preserving mucosal homeostasis in AADs (37, 38). The integrity of this barrier is governed by the interaction between tight junctions, cellular oxidative stress responses, and m6A regulatory pathways. Emerging evidence suggests that reciprocal regulation between m6A methyltransferases and non-coding RNAs constitutes one of the mechanisms governing airway epithelial barrier integrity. They exert bidirectional regulatory effects on epithelial barrier integrity in AR through interactions with non-coding RNAs. Zhang et al. (39) discovered that the long non-coding RNA FOXD3-AS1 is notably reduced in the nasal mucosa of AR patients. By binding to the RNA-binding protein RBM15B, FOXD3-AS1 recruits the METTL3/METTL14 methyltransferase complex, promoting the m6A modification of the pro-apoptotic factor CHOP mRNA and accelerating its degradation. This process inhibits the PERK-eIF2α-ATF4-mediated endoplasmic reticulum stress pathway, thereby restoring the expression of tight junction proteins Occludin and ZO-1. This suggests that METTL3 is involved in maintaining the integrity of the AR epithelial barrier. Conversely, METTL3 also stabilizes the circular RNAs circMIRLET7BHG and circCDKAL1 in an m6A-dependent manner, thereby exacerbating epithelial barrier dysfunction in AR. Following the YTHDC1-mediated nuclear-cytoplasmic transport, circMIRLET7BHG is recognized by IGF2BP1, which enhances the stability of ADAM10 mRNA (40). This process results in the downregulation of tight junction protein expression and an increase in epithelial permeability. Conversely, circCDKAL1 enhances IGF2BP2-dependent degradation of JARID2, thereby increasing HMGB1 expression and contributing to epithelial thickening, goblet cell hyperplasia, and nasal mucosal inflammation (41). Collectively, these findings highlight the complexity of reciprocal regulation between m6A methylation and non-coding RNAs.

Additionally, interactions within the epigenetic-immune-oxidative stress axis also contribute to epithelial barrier disruption. Persistent oxidative stress accelerates the degradation of GPX4 protein, leading to lipid peroxide accumulation and ferroptosis. The molecular patterns linked to damage that are released during ferroptosis enhance the inflammatory response, perpetuating a cycle of inflammation and oxidative damage. In this context, METTL3 modulates the PTBP1–TXNIP axis through m6A modification, resulting in decreased GPX4 levels, which in turn heightens susceptibility to lipid peroxide accumulation and ferroptosis (42). This process contributes to the perpetuation of chronic inflammation and nasal mucosal damage in AR. However, METTL3 exerts an opposing effect in oxidative stress responses in AA. In bronchial epithelial cells, METTL3 expression is diminished and is associated with disease severity. METTL3 stabilizes GPX4 mRNA via m6A modification, thereby inhibiting ferroptosis and promoting epithelial cell viability (43). These findings indicate that the biological function of METTL3 is contingent not only on its catalytic activity as an m6A methyltransferase but also on the epithelial microenvironment and its downstream targets. Beyond METTL3, other m6A regulatory factors are implicated in this regulatory network. For instance, studies have demonstrated that in conditional WTAP knockout AR mice, a reduction in m6A methylation facilitates the translation of FBXW7 mRNA (44). FBXW7, part of the SKP1–CUL1–F-box E3 ubiquitin ligase complex, is essential for controlling various biological functions such as cell proliferation, survival, DNA repair, and oxidative stress (45–47). The upregulation of FBXW7 has been demonstrated to restore epithelial cell viability by reducing oxidative stress damage caused by IL-13 in nasal epithelial cells (NECs) (44). The research by Xiong et al. (48) demonstrated that oxidative stress induced by PM2.5 exposure upregulated FTO expression, leading to m6A demethylation of IKBKB mRNA and increased mRNA stability, thereby enhancing NF-κB signaling. This process results in a marked reduction in the expression levels of three critical tight junction proteins. Consequently, METTL3, WTAP, and FTO might form a complex network that disrupts epithelial homeostasis by engaging in oxidative stress processes within the AADs epithelium.

Notably, the impact of FTO on epithelial barrier integrity extends beyond its involvement in oxidative stress and also encompasses the proper differentiation and functional maturation of motile cilia. In an ovalbumin-induced AA model, FTO deficiency accelerates Foxj1 transcript degradation, resulting in impaired ciliogenesis and disrupted epithelial homeostasis (49). This underscores the multiple roles of FTO in preserving airway epithelial integrity.

#### 4.1.2 Inflammatory initiation and amplification

The airway epithelium not only serves as a sensor for environmental stimuli but also acts as an effector that triggers and amplifies inflammatory cascades. Among the detrimental environmental factors, airborne fine particulate matter (PM2.5) is strongly connected to the onset and exacerbation of AADs. Emerging evidence further implicates multiple m6A regulators as potential molecular biomarkers of PM2.5-induced asthma. Specifically, YTHDF2 stabilizes the expression of STC2 mRNA in a METTL3-dependent manner (50). STC2 is a glycoprotein expressed in multiple tumor tissues that promotes cellular adaptation to stress and inhibits apoptosis (51, 52). In airway epithelial cells, STC2 enhances SQSTM1 expression by preventing its ubiquitination and subsequent proteasomal degradation, thereby inducing mitochondrial autophagy. PM2.5 exposure markedly activates this pathway, thereby promoting airway inflammation and exacerbating asthma. Additionally, STC2 levels exhibit notable seasonal variation, with a significant increase during winter, which closely correlates with the temporal distribution of PM2.5 exposure. This correlation suggests the potential of STC2 as an environment-related biomarker and a prospective therapeutic target (50). Moreover, PM2.5 exposure can also stimulate epithelial cells to release exosomal lncRNA PAET, which stabilizes METTL3 and thereby promotes m6A modification of cytochrome c oxidase subunit 4 isoform 1 (COX4I1) (53). COX4I1, an isoform of cytochrome c-oxidase (complex IV of the respiratory chain), functions as the terminal electron acceptor in the mitochondrial respiratory chain and plays a critical role in regulating oxidative phosphorylation (OXPHOS) (54). The m6A-modified COX4I1 undergoes degradation upon recognition by YTHDF3, which subsequently disrupts epithelial OXPHOS, elevates DNA damage levels, and promotes the expression of markers indicative of airway remodeling (53). Polycyclic aromatic hydrocarbons (PAHs), major toxic components of PM2.5, also contribute to airway inflammation by modulating the expression of m6A regulatory factors. Mechanistically, polycyclic aromatic hydrocarbon exposure induces widespread FTO degradation and decreases global m6A levels in bronchial epithelial cells. FTO deficiency enhances the stability of ACKR3 mRNA in an m6A-dependent manner, thereby increasing ACKR3 expression. Elevated ACKR3 subsequently triggers autophagy and inflammatory responses by activating the TNF-α/NF-κB signaling pathway. Furthermore, knockdown of IGF2BP1–3 eliminated the stabilizing impact of FTO deficiency on ACKR3 mRNA, indicating that FTO cooperates with the IGF2BP family proteins in regulating ACKR3 mRNA methylation and stability (55). The findings might reveal new mechanistic perspectives on preventing and managing asthma triggered by environmental conditions.

Another characteristic of AADs is the pronounced circadian variation in symptom severity. Therefore, Wang et al. (56) investigated the interplay between core genes in circadian signaling pathways and m6A modifications. Their research demonstrated that, following house dust mite (HDM) stimulation, the YTHDF1 protein undergoes liquid-liquid phase separation and facilitates the recruitment of CLOCK mRNA to its YTH domain, thereby enhancing CLOCK translation. CLOCK subsequently activates the NLRP3 inflammasome and promotes IL-1β release, leading to airway inflammatory responses.

#### 4.1.3 Airway remodeling

Airway remodeling is a structural hallmark of AADs chronicity, with EMT as a central process, characterized by loss of E-cadherin expression, upregulation of N-cadherin and vimentin, and enhanced cellular migratory capacity (38, 57, 58). Under TGF-β1 stimulation, ETS1 and RBPJ cooperatively upregulate METTL14 expression. METTL14 suppresses EMT in bronchial epithelial cells via m6A-dependent mechanisms, preserving E-cadherin expression while reducing N-cadherin and vimentin levels, thereby attenuating airway remodeling (59). Additionally, the regulation of microRNAs through m6A modifications plays a significant role in the regulatory network governing airway remodeling. METTL3-mediated m6A modification stabilizes the expression of miR-143-3p, which directly targets and downregulates Smad3, further inhibiting the EMT process (60). In the HDM-induced AA mouse model, FTO was found to stabilize the specific transcripts encoding extracellular matrix proteins and adhesion molecules via its demethylase activity, thereby facilitating NF-κB activation and EMT (20). These studies emphasize the crucial role of m6A modification in maintaining the dynamic equilibrium of airway EMT, providing a theoretical framework for developing intervention strategies targeting m6A regulatory factor to address airway remodeling.

### 4.2 Innate immune cells

Innate immune cells, as fundamental components of allergic airway inflammation, play a critical role in directly defending against invading environmental molecules and initiating adaptive immune responses. This review focuses on four major innate immune cell types, including macrophages, dendritic cells, mast cells, and ILC2s, and summarizes m6A-mediated regulatory mechanisms underlying their roles in disease progression (see **Figure 2** and **Table 2**).

**Figure 2:**
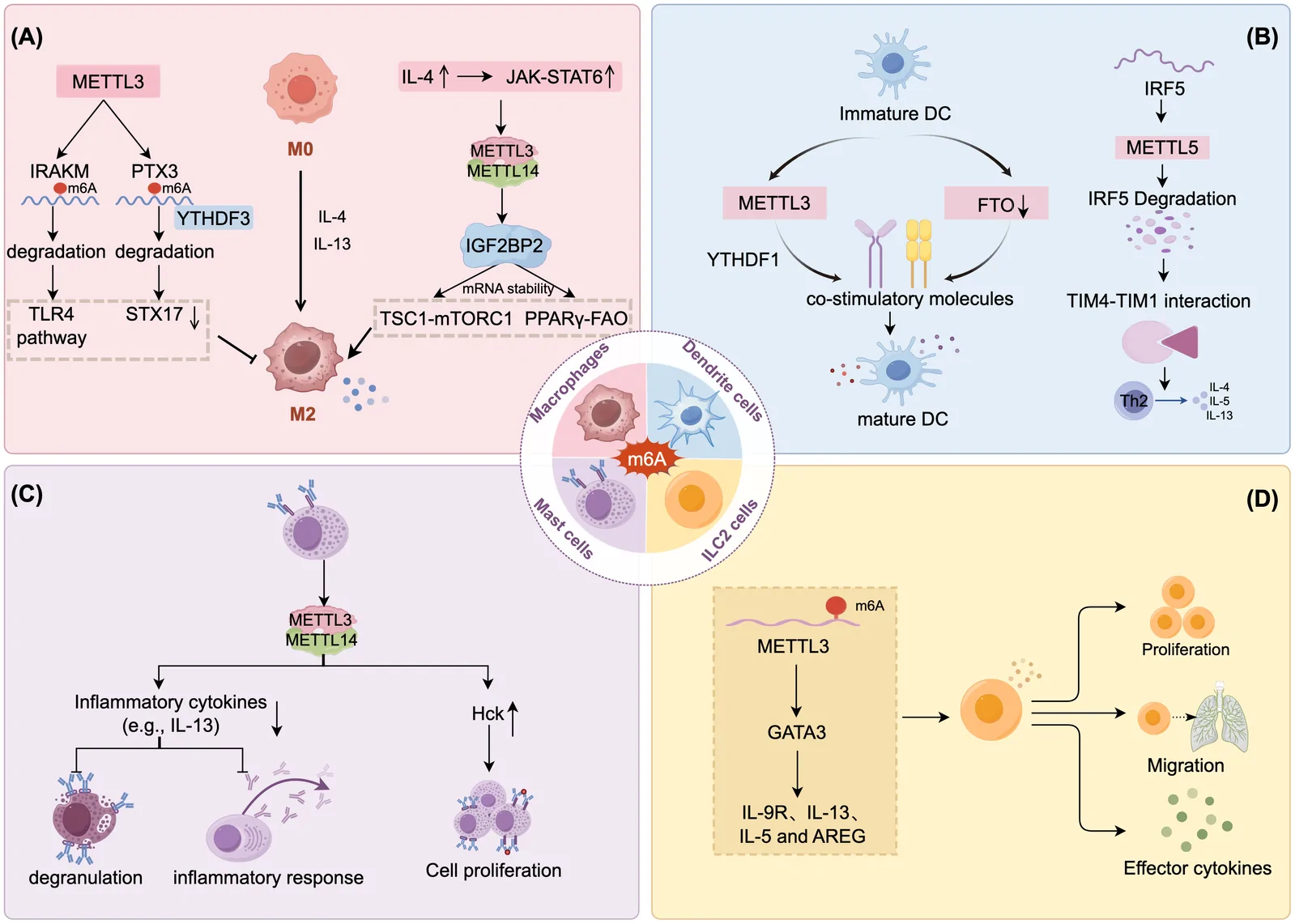
The role of m6A modification in innate immune cells. **(A–D)** Systematically delineate the dynamic regulatory mechanisms of m6A modification within macrophages, dendritic cells, mast cells, and ILC2s. IL-4, Interleukin-4; IL-5, Interleukin-5; IL-13, Interleukin-13; DC, dendritic cell; Th2, T helper type 2 cells; ILC2, group 2 innate lymphoid cells; IL-9R, Interleukin-9 receptor.

**Table 2:** The role of m6A modification in immune cells.

Study	Cell type	m6A regulator	Target gene / pathway	Biological function
(66)	Macrophage	METTL3	IRAKM	Inhibit M2 macrophage activation
(19)		METTL3 and YTHDF3	PTX3	Inhibit M2 macrophage activation
(67)		IGF2BP2	TSC1\PPARγ	Promote M2 macrophage activation
(71)	DC	METTL3 and YTHDF1	Transcripts of CD40, CD80, and the TLR4 adaptor protein Tirap	DC maturity
(79)		METTL5	IRF5	Th2 inflammatory response
(72)		METTL3	CD80 and CD86	DC maturity
(73)		FTO	CD80 and CD86	DC maturity
(87)	Mast cell	METTL3/METTL14	hck and IL-13	Maintaining cell growth and inhibiting inflammatory responses
(50)	ILC2	METTL3	GATA3	Enhanced proliferation, migration, and transcriptional activity of ILC2s
(53)	Naive T cell	METTL3	SOCS	T-cell proliferation and differentiation
(55)	Treg cell	METTL3	SOCS	Loss of Treg suppressive function
(56)	CD4+ T cell	METTL3 and YTHDF2	Foxp3	Reduced differentiation of CD4^+^ T cells into Treg cells
(59)		METTL3	miR-192-5p	Increased Th1 cell abundance and reduced Th2 and Th17 cell abundance, pulmonary inflammation, and T-cell differentiation
(60)		METTL3	Tcf	Tfh differentiation and germinal center reactions
(20)	pro-B cell	METTL14 and YTHDF2	IL-7 pathway	Pro-B cell proliferation and the transition from large pre-B cells to small pre-B cells
(112)	B cell	METTL14 and YTHDF2	Lax1 and Tipe2	Impaired GC B-cell proliferation and defective antibody responses
(113)		IGF2BP3 and YTHDF2	Myc and genes related to oxidative phosphorylation	Cell proliferation and oxidative phosphorylation
(114)		YTHDF2	Transcripts encoding transcriptional repressors and promoters of plasma cell differentiation	Plasma cell differentiation or survival

#### 4.2.1 Macrophages

Macrophages are pivotal in orchestrating both innate and adaptive immunity. Upon activation, they engulf pathogens and release cytokines, participating in various inflammatory activities. Macrophages can polarize into two distinct states, M1 and M2, which exert pro-inflammatory and anti-inflammatory effects, respectively (61). The predominance of M2 polarization is a significant driver of AADs progression (62–65). Extensive research has demonstrated that within the context of AA, METTL3 and YTHDF3 function as negative regulators of M2 polarization, whereas IGF2BP2 acts as a positive regulator.

Specifically, METTL3-mediated m6A modification targets early response genes, thereby expediting macrophage activation and initiating innate immune responses. In macrophages, the Toll-like receptor 4 (TLR4) signaling pathway primarily recognizes invading pathogens and induces the production of pro-inflammatory cytokines, including TNF-α and IL-6. Interleukin-1 receptor-associated kinase 3 (IRAK3), also known as IRAKM, acts as a negative regulator of TLR4 signaling, with its activity being directly influenced by m6A modification mediated by METTL3. METTL3 deficiency reduces m6A deposition on IRAKM mRNA and delays its degradation, resulting in IRAKM accumulation and subsequent suppression of TLR4 signaling in macrophages. This process is accompanied by increased expression of M2 macrophage-associated markers, including CD206, IL-10, and Arg1 (66). Similarly, upregulation of M2-associated genes has also been observed in alveolar macrophages from METTL3-deficient mice. PTX3, a positive regulator of M2 macrophage activation, is coordinately suppressed through METTL3- and YTHDF3-mediated m6A methylation (19). Collectively, these findings identify METTL3- and YTHDF3-driven m6A modification as a central mechanism governing macrophage activation.

Conversely, IGF2BP2 recognizes and stabilizes PPARγ and TSC1 transcripts in a manner dependent on m6A methylation. PPARγ plays a role in fatty acid oxidation and regulates the alternative activation of macrophages, while TSC1 serves as a negative regulator of the mTORC1 signaling pathway. The TSC1-mTORC1 pathway, along with PPARγ, facilitates the differentiation of M2 macrophages (67).

Collectively, IGF2BP2, METTL3, and YTHDF3 constitute a complex regulatory network involved in m6A methylation during macrophage polarization. Considering recent advancements in single-cell RNA sequencing (scRNA-seq) that have uncovered significant differences in the transcriptional profiles of macrophage subsets, it is imperative to further elucidate the role of m6A methylation in the heterogeneity and plasticity of these subsets.

#### 4.2.2 DCs

DCs, as antigen-presenting cells, are responsible for the continuous recognition, processing, and presentation of surface antigens (68). Typically, immature DCs induce immune tolerance to harmless components, whereas co-stimulatory molecules on mature DCs elicit T-cell immune responses by binding to ligands on various immune cells (69, 70). Therefore, whether DCs induce immune activation or tolerance depends on the maturation stage and differentiation of the DC subpopulation. Emerging evidence indicates that mRNA m6A modification also undergoes dynamic changes during DC maturation, with mature DCs exhibiting significantly higher m6A levels than their immature counterparts (71). Upon inhibition of m6A modification, both DC activation and T-cell proliferation are impaired. Mechanistically, METTL3 depletion in DCs results in reduced expression of co-stimulatory molecules (CD40, CD80, CD86) and attenuation of the TLR4/NF-κB signaling pathway, ultimately leading to impaired DC phenotype and maturation (71, 72). Conversely, pharmacological inhibition of FTO (e.g., FB23-2) enhances the expression of CD80 and CD86, thereby promoting antigen presentation and DC maturation (73). Collectively, these findings establish m6A modification as a critical regulator of DC functional competence.

Beyond antigen presentation, DC maturation critically shapes the Th1/Th2 immune balance during airway inflammation. DC-derived interleukin-12 (IL-12) drives naïve CD4^+^ T cells toward a Th1 phenotype (74, 75), whereas T-cell immunoglobulin and mucin domain-containing protein 4 (TIM4) promotes Th2 differentiation (76–78). Notably, METTL5-mediated m6A modification facilitates the degradation of IRF5 in DCs, thereby indirectly upregulating TIM4 expression (79–81). TIM4 subsequently interacts with TIM1 on activated CD4^+^ T cells to promote Th2 polarization (76, 82, 83). Conditional deletion of METTL5 in DCs reverses Th2 polarization and ameliorates experimental AA, underscoring the pivotal role of m6A in DC-driven immune polarization.

#### 4.2.3 Mast cells

Mast cells contribute to type 2 immune responses by releasing inflammatory mediators and interacting with T and B cells (84, 85). The precise regulation of mast cell differentiation and function is crucial for enabling rapid responses to allergens while minimizing excessive host damage from the inflammatory response (86). Notably, mast cells deficient in METTL3 exhibit a significantly enhanced response to acute stimulation. Mechanistically, METTL3 deficiency markedly increases the stability of inflammatory transcripts, particularly IL-13 mRNA, thereby augmenting mast cell degranulation and responsiveness to IgE stimulation (87). Simultaneously, METTL3 deficiency impedes mast cell proliferation through downregulation of Hck, a Src family kinase essential for mast cell activation and expansion. Given that METTL3 typically functions as part of a methyltransferase complex with METTL14, the researchers employed CRISPR-Cas9 technology to specifically knock out the METTL14 gene in mast cells. They observed that METTL14 deficiency similarly results in reduced mast cell proliferation and viability, alongside increased cytokine expression (87). These findings collectively highlight that the integrity of the METTL3/METTL14 methyltransferase complex is essential for maintaining mast cell homeostasis and ensuring rapid reactions to stimuli.

#### 4.2.4 ILC2s

ILC2s are tissue-resident immune cells that are involved in the early initiation of allergic airway inflammation (88–91). The transcription factor GATA3 is crucial for the development, differentiation, and functional gene expression of ILC2s (89, 92, 93). GATA3 mRNA exhibits high levels of m6A modification in ILC2s. METTL3-mediated m6A deposition enhances GATA3 transcript stability (94). In contrast, METTL3 deficiency leads to reduced GATA3 expression, which consequently results in a marked reduction in the proliferation, migration, and effector cytokine production of ILC2s.

### 4.3 Adaptive immune cells

Adaptive immune cells, which primarily consist of various T lymphocyte subtypes and B cells, are fundamental to maintaining the equilibrium between tissue damage and repair signals. m6A-mediated regulation has emerged as a key epitranscriptomic mechanism governing the differentiation and activation of adaptive immune cells. Deciphering these regulatory networks will be instrumental in advancing the clinical translation of m6A-targeted interventions (see **Figure 3** and **Table 2**).

**Figure 3:**
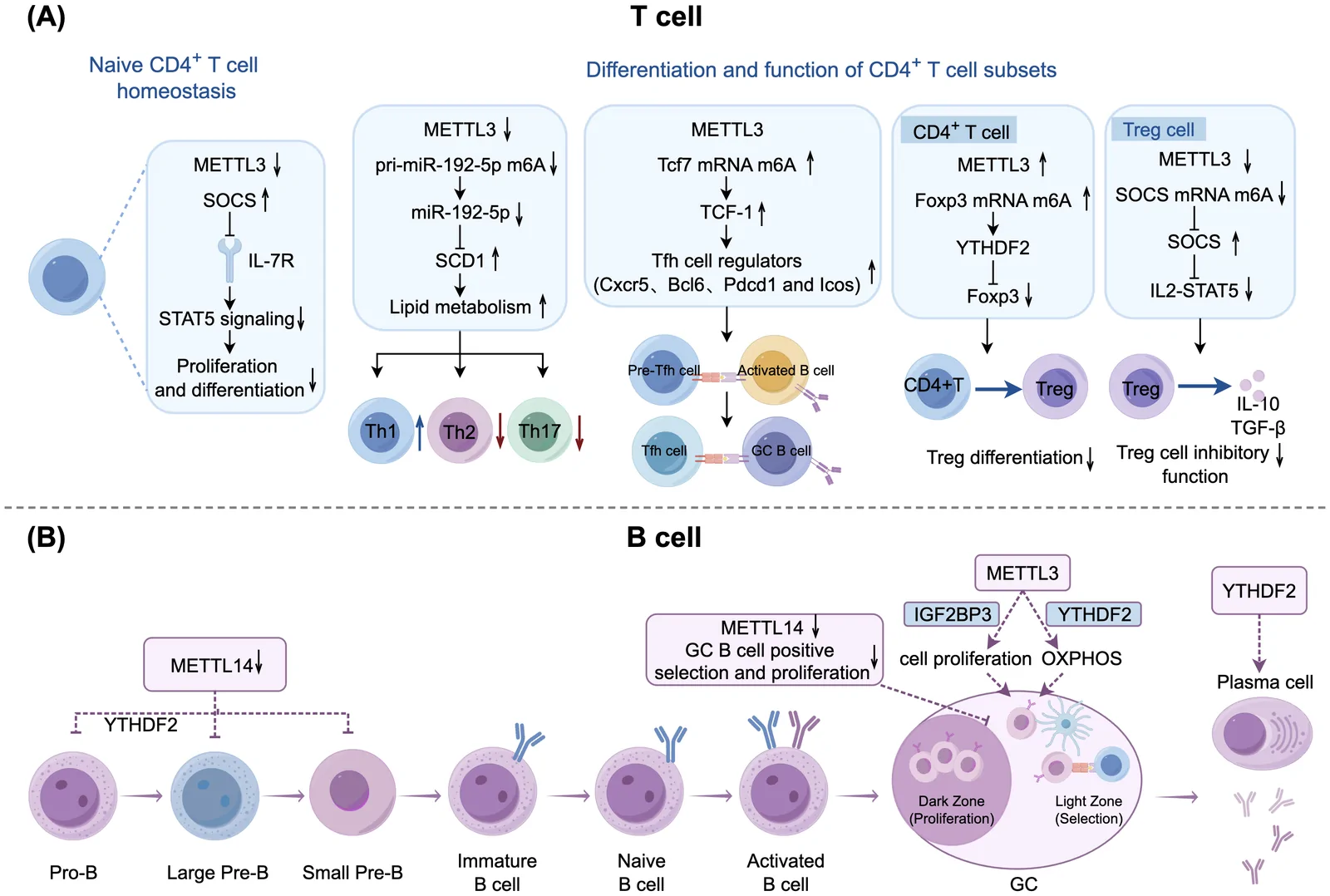
The role of m6A modification in adaptive immune cells. **(A)** This figure depicts the effect of m6A modification on the functionality of diverse T cell subsets, encompassing naïve CD4+ T cells, Th1, Th2, Th17, Tfh, and Treg cells. **(B)** This figure illustrates the impact of m6A modification on the maturation and development of B cells. Th1, T helper type 1 cells; Th17, T helper type 17 cells; Tfh, follicular helper T cells; Treg, regulatory T cells; GC, germinal center; OXPHOS, oxidative phosphorylation.

#### 4.3.1 T cells

T cells are pivotal in adaptive immunity, shaping inflammatory responses through cytokine-driven signaling while coordinating intercellular immune networks. Among these, CD4^+^ T cells are central to immune responses and demonstrate significant plasticity. Upon antigen encounter, naive CD4^+^ T cells differentiate into different subsets, including Th1, Th2, follicular helper T cells (Tfh), and regulatory T cells (Tregs), under the influence of distinct activation signals (95, 96). These Th cell subsets and their signature cytokines act individually or collectively to drive the pathogenesis of allergic airway diseases. Recent research reveals that m6A modification acts as a significant epigenetic regulator in determining the fate of CD4^+^ T cells, acting across multiple layers from initial T cell homeostasis to lineage differentiation and functional stabilization (97, 98).

During the early stages of T cell fate determination, m6A modification regulates naive CD4^+^ T cells homeostasis. In naïve T cells lacking METTL3, SOCS family activity is enhanced. SOCS proteins competitively bind to the IL-7 receptor, thereby inhibiting STAT signaling and its downstream pathways, ultimately hindering T cell proliferation and differentiation (99). Importantly, the asthma-associated genetic variant rs3194051 has been identified as an m6A-QTL in lung tissue and shows a significant association with IL-7 expression (100). This finding suggests that genetic variants may affect T cell differentiation and susceptibility to airway inflammation by modulating m6A-dependent IL-7 signaling pathways.

Beyond naïve T cells, m6A modification is indispensable for Treg differentiation and suppressive function. METTL3 deficiency leads to aberrant upregulation of SOCS proteins, which in turn inhibits the IL-2/STAT5 pathway critical for Treg lineage stability (101). Moreover, METTL3 regulates Foxp3 expression in a YTHDF2-dependent manner at the post-transcriptional level. Reduced Foxp3 expression impairs Treg differentiation, allowing pro-inflammatory subsets such as Th2 and Th17 cells to expand (102).

m6A modification also governs effector T-cell differentiation through metabolic reprogramming. The lipid metabolism–epitranscriptome axis has emerged as a key determinant of Th-cell polarization. Stearoyl-CoA desaturase 1 (SCD1), a rate-limiting enzyme in lipid synthesis (103), is essential for T-cell survival (104). Conditional deletion of METTL3 in CD4^+^ T cells reduces miR-192-5p expression, leading to increased SCD1 levels. This metabolic shift promotes Th1 differentiation while suppressing Th2 and Th17 lineages (105), highlighting a potential therapeutic axis linking m6A regulation to cellular metabolism. This connection offers the possibility that restoring miR-192-5p levels or pharmacologically inhibiting SCD1 could counteract this lineage bias and mitigate airway inflammation.

As specialized effector CD4^+^ T cells, Tfh cells are tightly regulated by m6A modification. METTL3 modulates key Tfh-associated genes, including ICOS and Tcf7, thereby suppressing the development and differentiation of Tfh cells (106). Importantly, Tfh cells exhibit functional duality in AADs: they promote allergen-specific IgE production while also limiting persistent type 2 inflammation (107). Whether m6A modification governs this functional switch remains an important unresolved question.

#### 4.3.2 B cells

B cells are an essential part of the humoral immune response triggered by inhaled allergens. They not only participate in antigen presentation and T cell helper responses, but also drive the production of IgE antibodies through antibody class switching, affinity maturation, and plasma cell differentiation (108, 109). B-cell development and differentiation proceed through multiple stages, broadly including pro-B cells, immature B cells, mature B cells, activated B cells, and plasma cells (PCs) (110). In this series of cell fate determination and differentiation, m6A modification serves as a critical regulatory mechanism.

Early B-cell development can be further subdivided into the pro-B-cell, early large pre-B-cell, late large pre-B-cell, and small pre-B-cell stages. METTL14 regulates cell proliferation and cell-state transitions through distinct mechanisms. At the pro-B-cell stage, loss of METTL14 diminishes YTHDF2’s ability to recognize and bind its targets, thereby impairing YTHDF2-mediated transcript degradation. This leads to aberrant upregulation of a subset of transcripts that negatively regulate the cell cycle, severely hindering pro-B-cell proliferation. Besides controlling the cell cycle, METTL14 facilitates the shift from large pre-B cells to small pre-B cells. Notably, the METTL14-mediated m6A modification of Ikzf3, Spib, Bcl6, and Cd2 occurs independently of YTHDF2 or YTHDF1 (111).

The transformation of mature B cells into IgE-producing plasma cells is known to be tightly and intricately controlled, involving T-cell help, germinal center (GC) reactions, and/or extrafollicular responses, as well as antibody class switching (14). The GC reaction is characterized by clear functional partitions: the dark zone (DZ), which promotes B-cell proliferation and somatic hypermutation; and the light zone (LZ), which supports the selection of high-affinity GC B cells. In GC B cells, METTL14 and METTL3 cooperatively regulate cell proliferation. METTL14 indirectly enhances GC B-cell proliferation and positive selection by promoting the mRNA decay of negative immune regulators, including Lax1 and Tipe2 (112). By contrast, METTL3-mediated m6A modification influences Myc mRNA stability and its downstream pathways through IGF2BP3, thereby maintaining normal cell-cycle progression in GC B cells. Meanwhile, METTL3 can also indirectly regulate the expression of OXPHOS-related transcripts through the function of YTHDF2 (113). These observations suggest that METTL3 exerts distinct functions through interactions with different m6A reader proteins, ultimately sustaining an effective GC response.

A recent study further indicated that m6A modification may also influence terminal B-cell differentiation and plasma cell accumulation. To identify RBPs involved in the differentiation or survival of CD138^+^ PCs, Turner and colleagues (114) performed a CRISPR/Cas9-based knockout screen targeting 1, 213 RBPs. The results showed that loss of YTHDF2 inhibited the accumulation of CD138^+^ PCs, but did not affect IgE class switching *in vitro*. Additional studies are required to clarify the precise biological mechanism through which YTHDF2 modulates plasma cell accumulation during allergic airway inflammation.

In summary, these findings suggest that m6A modification exerts cell-type-specific functional effects at distinct stages of B-cell development through interactions with different m6A reader proteins. Despite these advances, the roles of m6A modification in other B-cell subsets, particularly AADs-related subsets such as memory B cells and regulatory B cells, remain poorly understood. Further elucidation of the dynamic roles of m6A in B-cell fate determination, sustained IgE production, and the maintenance of allergic memory will therefore be essential.

## 5 Cellular context shapes the functional heterogeneity of m6A regulation in AADs

The biological roles of m6A modifications in AADs are not solely determined by the intrinsic properties of m6A regulatory proteins. Instead, they are significantly influenced by the specific cell type, disease stage, and local microenvironment. Distinct transcriptional programs and effector functions characterize airway epithelial cells, innate immune cells, and adaptive immune cells, resulting in functional diversity for a single m6A regulator. The roles of METTL3 in various immune cells exemplify this diversity. In ILC2s, METTL3-mediated m6A deposition supports proliferation and effector cytokine production, thereby promoting type 2 inflammatory responses (94). In contrast, in Tregs, METTL3 sustains immunosuppressive function by regulating Foxp3 expression (102). Additionally, the expression and activity of m6A regulators are dependent on the disease and tissue context, leading to variable outcomes based on the specific setting. For example, METTL3 and ALKBH5 are upregulated in the nasal epithelial cells of patients with AR but downregulated in the bronchial epithelial cells of patients with AA (21, 40, 115, 116). The data imply that the functions of individual m6A regulators are not straightforwardly pro-inflammatory or anti-inflammatory, but are instead influenced by the specific cell type and tissue environment.

Furthermore, the effects of the same m6A regulatory protein can vary substantially depending on the disease stage and cellular context. For instance, during the development of the ciliated airway epithelium, FTO facilitates the formation and function of motile cilia, thereby maintaining epithelial homeostasis by stabilizing Foxj1 transcripts (49). Conversely, in the context of chronic airway inflammation, FTO predominantly enhances NF-κB activation and airway remodeling (20, 48). Interestingly, under polycyclic aromatic hydrocarbon exposure, FTO may negatively regulate TNF-α/NF-κB signaling in an IGF2BP-dependent manner (55). These findings further illustrate the context dependence of FTO function and suggest that its biological effects should be interpreted in relation to the combined influence of cell type, disease stage, and local environmental cues.

Collectively, multiple layers of regulation contribute to the context-dependent effects of m6A on mRNA fate. A detailed understanding of these complex regulatory mechanisms is essential for the development of future m6A-targeted interventions for AADs. The emergence of single-cell m6A detection technologies, such as picoMeRIP-seq, scDART-seq, and single-nucleus m6A cleavage and labeling techniques, has enabled recent studies to begin dissecting cell-type- and context-dependent patterns of m6A regulation. First, various transcription factors and RNA-binding proteins (RBPs) are expressed in a cell-type-specific manner, facilitating cell-type-specific m6A modifications through distinct RNA- and DNA-binding domains (117–119). Secondly, non-coding RNAs also participate in cell-specific m6A deposition. MicroRNAs (miRNAs) can affect the binding of m6A regulatory proteins to mRNAs containing miRNA target sites through sequence pairing, thereby modulating the abundance of m6A in these transcripts (120). Finally, the composition of the m6A writer complex, along with the diversity in binding patterns and interactions among different types of m6A readers, contributes to the functional heterogeneity of m6A modifications (121).

## 6 The role of m6A dysregulation in epithelial-immune crosstalk

The above findings reveal the role of m6A modification in regulating airway inflammation across epithelial cells, innate immune cells, and adaptive immune cells. Notably, a prevailing hypothesis highlights epithelial-derived cytokines, collectively termed alarmins, including thymic stromal lymphopoietin (TSLP), interleukin-25 (IL-25), and interleukin-33 (IL-33), as central regulators that bridge epithelial–immune communication and initiate the pathophysiological cascade of allergic airway diseases (5, 57, 122). Compared with healthy individuals, patients with AADs exhibit markedly elevated levels of IL-33, IL-25, and TSLP in the airway, which correlate with disease severity (123–126). Although these alarmins differ in their cellular sources and receptors, they function synergistically to initiate and amplify type 2 immune responses. When exposed to environmental factors like allergens or microbes, alterations in m6A modification within epithelial cells can disrupt epithelial barrier integrity, thereby reshaping the alarmin expression profile and subsequently influencing the recruitment and polarization of diverse immune cell populations. Conversely, immune cells can secrete cytokines that either amplify or suppress epithelial cell function. In this context, epithelial-derived alarmins and inflammatory mediators likely constitute a critical hub through which m6A orchestrates the complex and dynamic interactions between epithelial and immune compartments, ultimately contributing to allergic sensitization and airway inflammation.

To date, available evidence has primarily focused on the role of m6A modification in communication between airway epithelial cells and T cells. Chen et al. (21) performed gene transfection experiments with METTL3 and SOX5 in human bronchial epithelial (HBE) cells and then co-cultured these treated cells with human CD4^+^ T cells. They found that METTL3 silencing in HBE cells increased SOX5 expression, accompanied by enhanced Th2 cell differentiation and increased expression of GATA3 and IL-4, whereas Th1 cells and the expression of T-bet and IFN-γ were reduced. Conversely, METTL3 overexpression produced the opposite effects. Moreover, SOX5 silencing reversed the Th2-skewed response induced by METTL3 deficiency. These results suggest that METTL3 facilitates the m6A-dependent degradation of SOX5 mRNA in HBE cells, thereby suppressing Th2 cell differentiation. In addition, environmental exposure may also remodel the immune microenvironment by altering the m6A regulatory status of epithelial cells. In pediatric AA bronchial epithelial cells, PM2.5 exposure suppresses ALKBH5 expression and, in cooperation with YTHDF1, enhances m6A modification and stability of SRSF1 mRNA, ultimately aggravating Th1/Th2 imbalance in sensitized mice (127). Despite advances in research, the precise role of epithelial alarmins in mediating the Th1/Th2 imbalance in AA remains unclear, necessitating further investigation into how m6A regulation influences alarmin release and subsequent T-cell differentiation.

Zhan et al. (40) notably identified the regulation of epithelial alarmin expression via m6A modification within a murine model of AR. It has been observed that the overexpression of the METTL3 gene leads to a significant upregulation of IL-25, IL-33, and TSLP, while concurrently downregulating Th1 cytokines (such as IFN-γ) and upregulating Th2 cytokines (such as IL-4 and IL-5). While the study suggests that m6A modification may facilitate epithelial-immune interaction by modulating epithelial alarmins, the implications of m6A in this intercellular communication warrant cautious interpretation, given the lack of functional blockade or rescue experiments specifically targeting these alarmins.

Moreover, m6A modification plays a crucial role in modulating the response of epithelial cells to Th2 cytokines. IGF2BP3 modulates type 2 airway inflammatory responses by inhibiting IL-13/IL-4 receptor signaling. Silencing IGF2BP3 increases the expression of IL-4R and IL-13Rα1 on the cell surface, enhancing STAT6 phosphorylation and activity and facilitating the transition of primary airway epithelial cells to a type 2 phenotype (128).

However, research on the role of m6A in interactions between other immune cells and airway epithelial cells remains sparse. For example, Wang and colleagues demonstrated that YTHDF1 deficiency markedly reduces the expression of epithelial-derived alarmins, suggesting that YTHDF1 may play a role in regulating the signaling output from epithelial cells during immune responses (56). Nonetheless, the specific downstream target cells influenced by these alarmins and their precise immune effects remain poorly elucidated. KEGG pathway analysis showed that genes with elevated m6A modification in airway epithelial cells of allergic mice are predominantly enriched in B cell receptor signaling, cancer-related pathways, thyroid hormone signaling, and circadian rhythm pathways, providing potential insights for investigating m6A-mediated epithelial–B-cell communication. Furthermore, tumor necrosis factor-like cytokine 1A (TL1A) has recently emerged as a novel alarmin expressed in both human and murine bronchial epithelial cells (129). Accumulating evidence indicates that the TL1A/DR3 axis functions as a co-stimulatory factor for ILC2s (130–132). TL1A synergizes with IL-33 to enhance ILC2 activation and IL-9 production, thereby amplifying the type 2 inflammatory cascade (133). These findings point to the need for further investigation to determine whether m6A modification can be exploited to disrupt TL1A-driven alarmin signaling networks.

Nonetheless, current research predominantly uses animal models and *in vitro* cell cultures, which have inherent limitations in replicating the complexity of the human immune system, particularly with respect to epithelial–immune interactions (134). Organoids, self-organizing multicellular structures generated *in vitro*, can replicate the microscopic architecture and functions of real organs *in vivo*. The integration of organoid models with epitranscriptomic profiling will systematically elucidate the dynamic regulatory networks of m6A in epithelial–immune crosstalk.

## 7 Clinical applications of m6A

AR and AA encompass a diverse array of phenotypes characterized by distinct immunological properties and clinical outcomes. Although these phenotypes exhibit some similarities in clinical manifestations, triggering factors, and inflammatory processes, there are notable differences in their treatment responses and disease progression (135–138). Recent genetic studies have identified numerous gene loci associated with immune regulation, airway pathology, and drug response (139–142). Nonetheless, these genetic variations account for only a fraction of the disease’s genetic risk, thereby limiting the advancement of targeted therapeutics and the enhancement of drug efficacy. The “missing heritability” may be attributed to transcriptional and post-transcriptional regulatory mechanisms that have not been fully accounted for (143, 144). Given the fundamental role of m6A modifications in gene expression regulations and its widespread dysregulation in AADs, drug development targeting m6A regulatory mechanisms has emerged as a focal point of current research. This section elucidates the potential applications of m6A modification in disease prediction and subtyping and provides a summary of the research progress on drugs targeting m6A regulatory factors in epithelial and immune cells.

### 7.1 The role of m6A modification in disease diagnosis

Accumulating evidence indicates that characteristic alterations in m6A methylation are closely associated with asthma susceptibility, disease severity, and immune heterogeneity. Peripheral blood m6A regulators have emerged as promising biomarkers for predicting childhood asthma risk (145). YTHDF3 and YTHDC1 have been identified as key regulators that can distinguish patients with severe asthma from healthy controls (146). Notably, m6A regulatory patterns correlate closely with immune-cell infiltration signatures, particularly those involving Th2 cells, mast cells, eosinophils, and macrophages. For instance, eosinophil abundance is positively associated with YTHDF3 expression but negatively associated with EIF3B expression (146); macrophage and Th2-cell infiltration inversely correlate with METTL16 expression (147). The results indicate that m6A methylation characteristics may mirror the underlying immune microenvironment, thereby contributing to risk stratification and the classification of disease endotypes.

### 7.2 The role of m6A modification in treatment

#### 7.2.1 Targeting m6A methyltransferase

As the most extensively investigated m6A methyltransferase, METTL3 has become a key player in regulating allergic airway inflammation, and its activity can be modulated by both sex hormones and the gut microbiota to exert anti-inflammatory effects. Chen et al. (148) have demonstrated that androgen intervention can mitigate airway inflammation in AA by specifically modulating METTL3 in bronchial epithelial cells. Mechanistically, METTL3 deficiency alters the m6A methylation landscape, resulting in increased levels of GATA3, IL-4, and Th2 cells, alongside decreased levels of T-bet, IFN-γ, and Th1 cells. Dihydrotestosterone markedly reverses these effects, suppressing Th2 differentiation and airway hyperresponsiveness, whereas estradiol exerts opposing effects. Notably, sex dimorphism has been observed across a variety of immune cell populations implicated in AADs pathogenesis. However, current evidence regarding the role of m6A regulation in mediating these sex-specific effects is primarily limited to airway epithelial cells, with limited research on allergic rhinitis models or other immune cell subsets (149). Therefore, understanding the relationship between sex dimorphism and m6A methylation could aid in discovering new therapeutic targets. Furthermore, environmental stimuli originating from the microbiota and their metabolites can reshape the epigenetic landscape through diverse mechanisms. For instance, Saccharomyces boulardii can influence m6A modification levels in AA mice, particularly the expression of METTL3. By upregulating METTL3 in epithelial cells, Saccharomyces boulardii enhances gut microbiota diversity and metabolic homeostasis, and ameliorates inflammation and oxidative stress in an m6A-dependent mechanism (150). This evidence underscores the intricate connection between gut microbiota and pulmonary inflammation, suggesting that microbiota-targeted epitranscriptomic interventions may represent a novel therapeutic avenue, although their efficacy and safety require further validation.

In addition to METTL3, WTAP, an essential part of the m6A methyltransferase complex, demonstrates significant therapeutic potential. Astragalus polysaccharides (APS) natural bioactive compounds extracted from Astragalus (151, 152), have been shown to inhibit WTAP expression across various cell types, thereby exerting an anti-inflammatory effect. For example, APS-mediated WTAP inhibition alleviates IL-13-induced oxidative stress injury in human NECs (44). In THP-1 macrophages, APS modulates IL-6 expression through WTAP-dependent m6A regulation, thereby attenuating inflammatory responses (153).

Beyond natural products, several newly developed small-molecule inhibitors have also been reported to influence WTAP transcription and expression. Previous studies have revealed that NF-κB p65 directly regulates WTAP transcription, leading to marked WTAP upregulation in multiple inflammatory cell types, including bone marrow-derived macrophages, peripheral blood mononuclear cells, and THP-1 cells. Elevated WTAP levels further promote spontaneous liquid–liquid phase separation, thereby enhancing the interaction between the m6A methyltransferase complex and nuclear inflammatory transcripts, including IL6ST, IL18R1, and IL15RA, ultimately amplifying inflammatory responses. The NF-κB p65 inhibitor PG490 suppresses WTAP expression and reduces m6A abundance globally, partially reversing hyperinflammatory phenotypes (154). Collectively, these findings suggest that targeting WTAP represents a potential epitranscriptomic strategy to reprogram the intracellular m6A landscape and mitigate hyperinflammatory responses.

#### 7.2.2 Targeting m6A reader proteins

Tegaserod, a drug previously used for constipation (155), has been identified as a potential YTHDF1 inhibitor. Exposure to toluene diisocyanate upregulates YTHDF1 in human bronchial epithelial cells, intensifying mitochondrial dysfunction and airway inflammation in mice. Therefore, pharmacological inhibition of YTHDF1 may represent a promising therapeutic strategy for alleviating airway inflammation. However, tegaserod exhibits dose-dependent dual effects: low doses (1 mg/kg) attenuate airway hyperresponsiveness and mitochondrial dysfunction, whereas higher doses produce opposite outcomes. This phenomenon is attributed to the dose-dependent action of tegaserod, which influences not only its therapeutic effectiveness but also the overall m6A modification levels (156). These outcomes highlight the necessity for optimized dosing strategies or the development of more selective YTHDF1 inhibitors.

#### 7.2.3 Targeting m6A demethylases

FTO, an m6A demethylase, regulates multiple cellular processes in epithelial cells, such as cell proliferation, the TNF-α signaling pathway, and the EMT pathway. Currently, several small-molecule FTO inhibitors have been developed, such as emodin, meclofenamic acid, FB23, and FB23-2 (157–160). Among these, FB23 is a potent and selective inhibitor that targets FTO’s catalytic site. In BEAS-2B cells and primary human and murine epithelial cells, FB23’s ability to inhibit cell proliferation depends on the dose (20), a finding that contrasts with previous studies in acute myeloid leukemia cell lines (160). The observation implies that FB23 influences cell proliferation in a cell-type-specific manner. Moreover, increased m6A modifications were detected in Cadherin-3 and extracellular matrix components (ADAMTS6 and TGM2) in FB23-treated BEAS-2B cells, indicating that FB23 modulates the EMT pathway by inhibiting FTO demethylase activity (20). FTO is not only expressed in ciliated epithelial cell subpopulations but also broadly in smooth muscle and submucosal gland cells, making the lung cell types targeted by FB23 unclear. Consequently, further exploration is imperative to clarify the role of FTO in other airway cells to evaluate the safety and efficacy of FB23 as a treatment for AA.

#### 7.2.4 Other drugs targeting m6A regulatory factors

Beyond agents targeting individual m6A regulators, resveratrol has attracted increasing attention owing to its potential to modulate multiple components of the m6A regulatory network simultaneously. Resveratrol is a natural phytoalexin belonging to the stilbene class of polyphenolic compounds. Through integrated bioinformatic analyses of Gene Expression Omnibus datasets, six m6A-related differentially expressed genes, including FTO, IGF2BP2, RBM15, RBMX, WTAP, and YTHDC1, were identified as potential therapeutic targets of resveratrol in AA (161). However, further investigation is necessary to fully clarify the regulatory mechanisms governing m6A methylation. Nevertheless, current evidence is largely based on bioinformatic predictions, and more mechanistic and experimental research is needed to confirm the direct interactions between resveratrol and m6A regulators.

At present, the clinical translation of RNA modification-targeted therapies remains in its infancy. Advancements in CRISPR/Cas9, RNA interference, and nanocarrier delivery technologies have unveiled new opportunities for targeting these regulatory factors. Considering the widespread involvement of RNA modifications in basic physiological functions, the development of cell-type-specific and temporally controlled therapeutic strategies will be essential to transform RNA modifications from static biomarkers into dynamically regulatable determinants of immune behavior. In conclusion, these advancements create the foundational concepts for precision epitranscriptomic immunotherapy and might introduce new strategies for managing allergic airway diseases.

### 7.3 Efficacy evaluation of allergen-specific immunotherapy

AIT represents the sole therapeutic approach that directly addresses the underlying cause of AR by promoting immune tolerance rather than merely mitigating symptoms. Numerous studies have demonstrated that AIT can cause significant epitranscriptomic alterations in NECs. Consequently, evaluating changes in m6A modifications following AIT is essential for assessing treatment efficacy. Lai et al. (162) performed a comprehensive analysis of the m6A methylome and transcriptome in nasal epithelial cells from HDM-sensitized AR patients, highlighting the impact of m6A modifications on the formation of allergen-specific immune tolerance. This investigation identified 1, 455 hypermethylated and 4, 636 hypomethylated differentially expressed mRNAs, alongside 267 hypermethylated and 799 hypomethylated differentially expressed long non-coding RNAs (lncRNAs) post-AIT treatment. Notably, four mRNAs (KANK2, DBI, TNFRSF10A, and IL18) and three lncRNAs (SNHG14, LUCAT1, and AC105219) were correlated with the Total Nasal Symptom Score in AR patients, suggesting their potential as biomarkers for monitoring AIT efficacy. Moreover, integrated multi-omics analysis and Gene Set Enrichment Analysis demonstrated a marked downregulation of inflammation-associated pathways, notably the PI3K-AKT, MAPK, and Ras signaling pathways, alongside a significant activation of apoptosis and adhesion-related pathways following AIT. This indicates a transition towards epithelial repair and immune homeostasis. In conclusion, these findings imply that the m6A modification profile in NECs holds potential as a biomarker for predicting and monitoring the clinical efficacy of AIT.

## 8 Conclusion

AADs are chronic inflammatory disorders characterized by functional dysregulation across multiple cell types. In airway epithelial cells, m6A modifications regulate epithelial barrier integrity through diverse mechanisms involving non-coding RNAs, oxidative stress, ferroptosis, and mitochondrial homeostasis. They also modulate inflammatory signaling and EMT, thereby contributing to the amplification and persistence of airway inflammation. In immune cells, m6A primarily regulates the activation, differentiation, and effector functions of innate and adaptive immune populations, thereby promoting or restraining inflammation. Importantly, the effects of m6A regulators are highly dependent on cell type, tissue compartment, environmental exposure, and disease stage. Collectively, these findings suggest that m6A contributes to the pathogenesis and progression of AADs by coordinating epithelial dysfunction, immune dysregulation, and airway structural remodeling across distinct cellular contexts.

Currently, the cellular and spatial distribution of m6A in human allergic airways remains insufficiently characterized. Emerging single-cell epitranscriptomic approaches, such as scDART-seq (163), picogram-scale m6A RNA immunoprecipitation sequencing (164), m6A-isoSC-seq (165), and Nanopore direct RNA sequencing (166), enable the interrogation of m6A modifications at single-cell resolution. Notably, the regulation mediated by m6A modification is highly context-dependent: even the same m6A regulator targeting identical transcripts may exert opposing biological outcomes depending on tissue context or disease stage. Therefore, mapping a high-resolution single-cell, spatial, and cellular microenvironment of AADs will be essential for deciphering the mechanisms underlying the initiation and persistence of allergic inflammation, and for guiding the development of cell-selective or context-adapted therapeutic strategies.

Nonetheless, these advancements are insufficient for the clinical translation of AADs management. First, challenges such as drug delivery and off-target effects continue to impede the clinical application of epigenetic drugs (167, 168). Drug delivery systems using nanoparticles designed for particular tissues have the potential to enhance treatment efficacy and minimize side effects. For example, inhalable lipid nanoparticle systems have proven effective in delivering siRNA that targets epigenetic enzymes like HDAC9, thereby alleviating airway inflammation (169). Moreover, robust evaluation frameworks are needed to assess therapeutic efficacy. The targeted effects of m6A modulation can be systematically monitored using biomarkers, such as tissue-specific m6A RNA signatures. Beyond direct targeting of m6A regulators, modulation of cellular metabolism associated with m6A represents an additional therapeutic avenue. Metabolic reprogramming processes, including oxidative stress, autophagy, and apoptosis, can modulate trained immunity by inhibiting or activating a range of enzymes and signaling pathways that are essential for m6A modification dynamics (170, 171). The integration of single-cell sequencing and CRISPR-based approaches to construct cell-type-specific m6A metabolic regulatory networks will facilitate the identification of therapeutic targets.

In summary, further research is warranted to clarify the role of m6A modification in restoring airway immune homeostasis and to evaluate its potential as a therapeutic target for AADs.
